# Persistent Frustration-Induced Reconfigurations of Brain Networks Predict Individual Differences in Irritability

**DOI:** 10.1016/j.jaac.2022.11.009

**Published:** 2022-12-21

**Authors:** Julia O. Linke, Simone P. Haller, Ellie P. Xu, Lynn T. Nguyen, Amanda E. Chue, Christian Botz-Zapp, Olga Revzina, Samantha Perlstein, Andrew J. Ross, Wan-Ling Tseng, Philip Shaw, Melissa A. Brotman, Daniel S. Pine, Stephen J. Gotts, Ellen Leibenluft, Katharina Kircanski

**Affiliations:** Drs. Linke, Haller, Chue, Brotman, Pine, Leibenluft, and Kircanski, Mss. Xu, Nguyen, Revzina, and Perlstein, and Messrs. Botz-Zapp and Ross are with Emotion and Development Branch, National Institute of Mental Health, National Institutes of Health, Bethesda, Maryland. Prof. Tseng is with Yale Child Study Center, Yale School of Medicine, Yale University, New Haven, Connecticut. Dr. Shaw is with the Neurobehavioral Clinical Research Section, Social and Behavioral Research Branch, National Human Genome Research Institute, Bethesda, Maryland. Dr. Gotts is with the Section on Cognitive Neuropsychology, Laboratory of Brain and Cognition, National Institute of Mental Health, National Institutes of Health, Bethesda, Maryland.

**Keywords:** fMRI, frustration, graph-theory, irritability, prediction

## Abstract

**Objective:**

Aberrant responses to frustration are central mechanisms of pediatric irritability, which is a common reason for psychiatric consultation and a risk factor for affective disorders and suicidality. This pilot study aimed to characterize brain network configuration during and after frustration and test whether characteristics of networks formed during or after frustration relate to irritability.

**Method:**

During functional magnetic resonance imaging, a transdiagnostic sample enriched for irritability (N = 66, mean = age 14.0 years, 50% female participants) completed a frustration-induction task flanked by pretask and posttask resting-state scans. We first tested whether and how the organization of brain regions (ie, nodes) into networks (ie, modules) changes during and after frustration. Then, using a train/test/held-out procedure, we aimed to predict past-week irritability from global efficiency (E_glob_) (ie, capacity for parallel information processing) of these modules.

**Results:**

Two modules present in the baseline pretask resting-state scan (one encompassing anterior default mode and temporolimbic regions and one consisting of frontoparietal regions) contributed most to brain circuit reorganization during and after frustration. Only E_glob_ of modules in the posttask resting-state scans (ie, after frustration) predicted irritability symptoms. Self-reported irritability was predicted by E_glob_ of a frontotemporal-limbic module. Parent-reported irritability was predicted by E_glob_ of ventral-prefrontal-subcortical and somatomotor-parietal modules.

**Conclusion:**

These pilot results suggest the importance of the postfrustration recovery period in the pathophysiology of irritability. E_glob_ in 3 specific posttask modules, involved in emotion processing, reward processing, or motor function, predicted irritability. These findings, if replicated, could represent specific intervention targets for irritability.

Irritability is a common reason for pediatric psychiatric consultation and a risk factor for adult psychopathology^1^ and suicidality.^2^ Aberrant responses to frustration are thought to be key mechanisms of irritability.^3^ Frustration is a complex emotional and motivational state that is associated with distributed brain regions. Brain network dynamics related to the emergence of, and recovery from, frustration are poorly understood; such knowledge could guide the development of targeted interventions for irritability. Here, we conducted a pilot study in a transdiagnostic sample enriched for youth experiencing clinically significant irritability. Specifically, we coupled functional magnetic resonance imaging (MRI) during a frustrating task with pretask and posttask resting-state MRI. This allowed us to characterize frustration as a dynamic evolving process across brain circuits and to probe the utility of brain network metrics for predicting irritability.

Frustration occurs when actions fail to yield an expected reward (ie, frustrative nonreward [FNR]).^4^ Three small studies have investigated brain activation during or after FNR in healthy adults. These studies found effects in widely distributed cortical and subcortical brain regions,^5–7^ highlighting the importance of circuit-based approaches. In 21 traumatized male participants, anger induction, a process closely related to frustration induction,^8^ was associated with increased amygdala–inferior frontal gyrus connectivity during a subsequent resting-state fMRI scan.^9^ This suggests the utility of studying the chronometry of FNR-induced brain network changes.

In youth, studies using frustration tasks have focused on clinical samples enriched for irritability. These studies associated irritability with aberrant activity^10–12^ and connectivity^12,13^ of widely distributed brain regions during FNR. In addition, 3 studies found associations of irritability with neural responses immediately after FNR (ie, during the trial following a frustrating trial)^12,14^ or with reduced youth-caregiver prefrontal synchrony following an FNR task.^15^ Together, these findings suggest the relevance of brain function during and after frustration in elucidating mechanisms of irritability.

To study brain responses to frustration, we used a graph theory approach, which allowed us to perform a brain-wide, circuitry-based analysis. Graph theory posits that the brain is a network organized into subnetworks or circuits (modules) consisting of nodes (brain regions) and edges (functional connectivity between regions).^16,17^ Studies using graph theory approaches demonstrate that the canonical modules identified during resting state (ie, default mode, frontoparietal) reconfigure during cognitive^18^ and emotional^19,20^ tasks. During reconfiguration, connectivity among nodes changes, causing the splitting and merging of modules. Such reconfiguration alters the efficiency of information processing within modules, increasing the organism’s ability to respond effectively to environmental stimuli and demands. Few studies have used graph theory to study brain network function during affective processing, but there is evidence that emotional stimuli can elicit shifts toward more integrated brain organization^19^ and that trait emotional expression is related to frontoparietal and default mode module efficiency.^20^

Our first aim was to study brain network reconfiguration in response to frustration. Thus, we compared brain network configuration before frustration (pretask resting-state condition), during frustration (4 conditions of the frustration induction task; see below), and after frustration (posttask resting-state condition). First, at a global brain-wide level, we calculated the modularity index *Q* in each condition. *Q* quantifies the extent to which brain modules are organized into a relatively segregated state, with few intermodular connections, vs a more integrated state (aim 1a). Next, using the variation of information (VIn) metric, we compared how modular composition (ie, the specific nodes comprising each module) changes among the baseline pretask resting state, the 4 conditions of the frustration task, and the posttask resting state. We also identified the modules that underwent the highest degree of reorganization (aim 1b). Our second aim was to test whether the efficiency of information processing (global efficiency [E_glob_], ie, capacity for parallel information processing) of modules identified before, during, and after frustration was related to irritability. To ensure robustness of this exploratory analysis, we combined our within-person frustration manipulation with a multivariate approach. Specifically, we divided the sample into a training dataset, to test the predictive value of module E_glob_ for child and parent ratings of irritability, and a held-out dataset, to probe the utility of the prediction model in previously unseen data (aim 2).

Given the scarcity of relevant literature, we did not posit specific hypotheses for this pilot study. However, considering the reliability of the frustration-induction task^21^ and previous reports of functional alterations in brain regions following frustration,^5–7,9–11,13,14^ we expected significant modular reconfiguration during and after the task. Similarly, based on previous findings associating irritability with brain function following frustrating feedback,^12,14,15^ we expected that associations might emerge between irritability and brain network characteristics present in the posttask resting state. We used a repeated, within-person design, a multivariate data-driven approach, and rigorous correction for multiple comparisons^22^ to address concerns regarding the replicability of brain-behavior relationships in partially exploratory studies such as this one.

## METHOD

### Participants

Participants were recruited from the Washington, DC, metropolitan area in the context of 3 ongoing studies (ClinicalTrials.gov Identifiers NCT02531893, NCT00025935, and NCT00018057). Recruitment strategies leveraged established relationships with local health care providers and schools and included advertising on Facebook and sending postcards to local households. Participants were 66 youth (33 female participants, mean [SD] age = 14.0 [2.8] years, range = 9.3–20.9), 48 of whom met criteria for one or more psychiatric disorders as established by a licensed clinician using the Schedule for Affective Disorders and Schizophrenia for School-Age Children–Present and Lifetime version (K-SADS-PL).^23^ Diagnoses included disruptive mood dysregulation disorder (n = 14) and oppositional defiant disorder (n = 4), for which irritability is a diagnostic criterion^24^; attention-deficit/hyperactivity disorder (n = 26); separation anxiety disorder (n = 6); social phobia (n = 9); and panic disorder (n = 3), in which irritability is also common.^25,26^ A total of 25 participants were taking psychotropic medication (antidepressants: n = 9; anticonvulsants: n = 3; antipsychotics: n = 3; stimulants: n = 13; nonstimulant attention-deficit/hyperactivity disorder medication: n = 3). For each participant, we calculated the composite measure of medication load,^27^ which we used as a nuisance variable in our analyses. Exclusion criteria were neurological disorders, autism spectrum disorder, psychosis, bipolar disorder, substance use, MRI contraindications, and Full Scale IQ <70. The sample comprised participants with different racial and ethnic backgrounds (White, non-Hispanic: n 30; White, Hispanic: n = 7; Black or African American, non-Hispanic: n = 11; multiple racial identities, non-Hispanic: n = 11; multiple racial identities, Hispanic: n = 5; American Indian or Alaska native, non-Hispanic: n = 1). The socioeconomic status^28^ of participants was widely distributed (mean [SD] = 39 [24], range = 20–120). Institutional review board approval, parent or guardian consent, and child assent were obtained.

### Procedures

**FIGURE 1:**
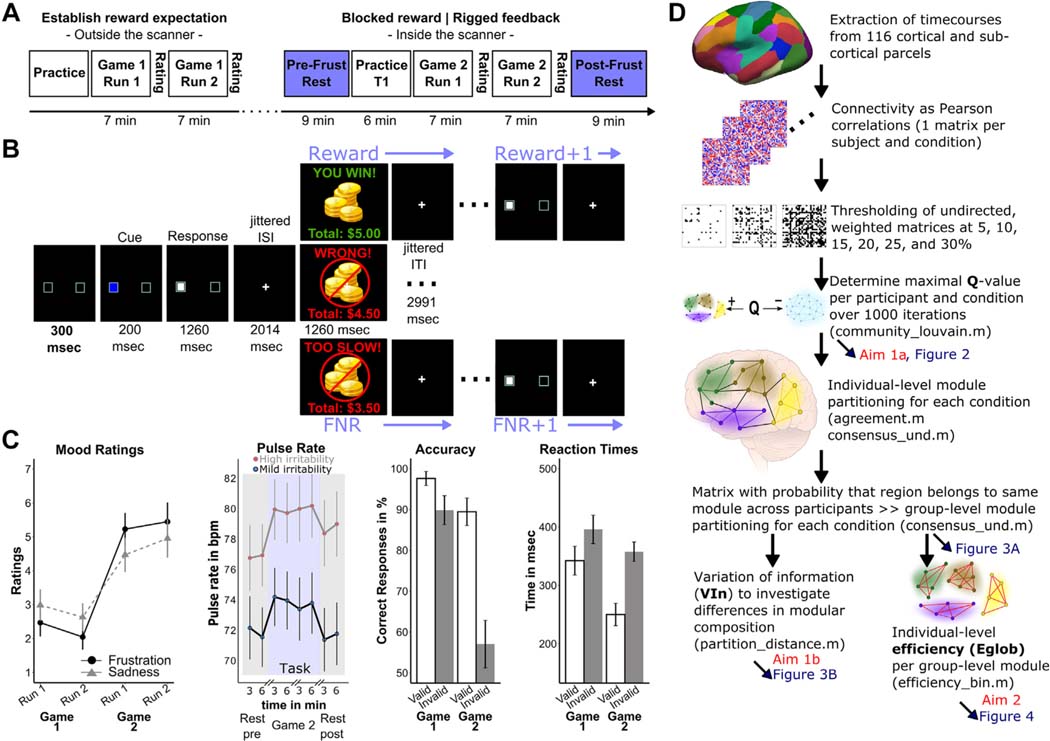
Experimental Design, Trial Sequence, Behavioral Results, and Processing Pipeline **Note:** (A) Overview of the paradigm. Game 1, conducted outside the scanner, established a reward expectation. Game 2 induced frustration during functional magnetic resonance imaging. Before and after game 2, 9 minutes of resting-state data were acquired. (B) Order and timing of 1 trial. Feedback can be FNR or Reward. FNR+1 denotes the anticipation phase on trial N+1, where the feedback on trial N was FNR. Similarly, Reward+1 denotes the anticipation phase on trial N+1, where the feedback on trial N was Reward. (C) Mood ratings, pulse rate, accuracy, and reaction time. Mood ratings (frustration and sadness) are shown during nonfrustrating, out-of-scanner game 1 and during frustrating, in-scanner game 2. Pulse rate was recorded during functional magnetic resonance imaging only. It is shown as a function of parent-rated irritability during the first and last half of pretask resting state, runs 1 and 2 of game 2, and the first and last half of posttask resting state. The Posner effect (lower accuracy and longer reaction times to invalid vs valid cues) across games 1 and 2 is also shown. Error bars represent 95% CI. (D) Overview of the processing pipeline. It shows the main processing steps: the acquisition of the 3 metrics of interest (Q, VIn, E_glob_) and how these metrics relate to study aims and figures. Scripts from the Brain Connectivity Toolbox that were used in the analysis are noted. Condition refers to the pretask and posttask resting state and the 4 task-based events of interest. bpm = beats per minute; E_glob_ = global efficiency; FNR = frustrative nonreward; Frust = frustration; ITI = intertrial interval; ISI = interstimulus interval; Q = modularity index Q; VIn = variation of information.

In the MRI scanner, participants completed a resting-state scan (pre-RS), followed by a frustration-inducing attention-orienting task, followed by another resting-state scan (post-RS) (Figure 1A).^10,14^

#### Resting-State Scans.

The pre- and post-RS functional MRI acquisitions were 9 minutes each. During the scans, participants viewed a fixation cross.

#### Frustration-Inducing Attention-Orienting Task.

The attention-orienting task was a modified Posner task, in which participants pressed a button as quickly as possible to indicate a target location (left or right side of screen) after a valid cue (75% of trials) or invalid cue (25% of trials). Before entering the scanner, participants completed an initial version of the task (game 1) to establish reward expectation. In game 1, correct responses (approximately 98% of trials) were rewarded with $0.50. After the pre-RS scan, to ease participants into the frustration induction while structural MRI was obtained, participants completed 32 task trials, with 4 randomized trials delivering rigged feedback in which $0.50 was deducted. This was followed by functional acquisition during the frustration-inducing version of the task (game 2; 2 runs of 50 trials each). In this version,^10,14^ after 60% of correct responses, $0.50 was deducted under the pretense that responses were too slow. This caused participants to lose a large portion of their previously accumulated winnings. After each run of game 1 and game 2, self-reported frustration and unhappiness were assessed using 9-point Likert scales (1 = “happy” or “not at all frustrated”; 9 = “sad” or “extremely frustrated”).

#### Additional Measures.

Before scanning, parents and youth independently rated child irritability during the past week using the Affective Reactivity Index (Figure S1, available online).^29^ On the task, participants’ reaction time and accuracy were measured for valid trials, in which the cue appeared in the same location as the target, and invalid trials, in which the cue appeared in the opposing location from the target; invalid trials are more cognitively demanding. Pulse rate from finger photoplethysmography^30^ was recorded throughout scanning to index physiological arousal.

### Processing of Imaging Data

Data were acquired on 2 identical 3.0T scanners. Data quality was assessed using the MRI Quality Control tool (MRIQC v0.15.2).^31^ Fourteen participants were excluded for motion-related artifacts (framewise displacement >0.5 mm/repetition time for >30% of the images). Data for the remaining 66 participants were preprocessed with fMRIPrep v20.0.5.^32^

We modeled brain network configuration during 6 conditions, including pre-RS, post-RS, and 4 task events in game 2: frustrating deduction of $0.50 (FNR); anticipation of the next trial’s feedback after FNR (FNR + 1); win of $0.50 (Reward); and anticipation of the next trial’s feedback after Reward (Reward+1) (Figure 1B). To do so, we constructed a functional connectivity network comprising 116 nodes: 100 cortical parcels assigned to known functional networks^33^ and 16 subcortical regions from the fMRIPrep FreeSurfer segmentation. We regressed out from the time series motion parameters, ICA-AROMA^34^ head motion components, white matter and cerebrospinal fluid signal, the first 3 principal components from aCompCor,^35^ the first 3 cosine variables, framewise displacement, and the spatial standard deviation of the temporal difference data; global signal was not regressed out. Task conditions convolved with a canonical hemodynamic response function were also regressed from the time series to remove variance associated with task-related coactivation.^36,37^ After accounting for the hemodynamic lag, for each node we created a time series specific to each game 2 task event (FNR, FNR+ 1, Reward, Reward +1) by concatenating the residual time series associated with the event across all relevant trials. Functional connectivity was quantified using Pearson correlations transformed for normality using Fisher $z^{\prime }$ transformation. This resulted in 6 connectivity matrices per subject: pre-RS, post-RS, and game 2 FNR, FNR+1, Reward, and Reward+1 (Figure 1D).

### Does Frustration Impact the Degree of Brain Network Segregation?

**FIGURE 2:**
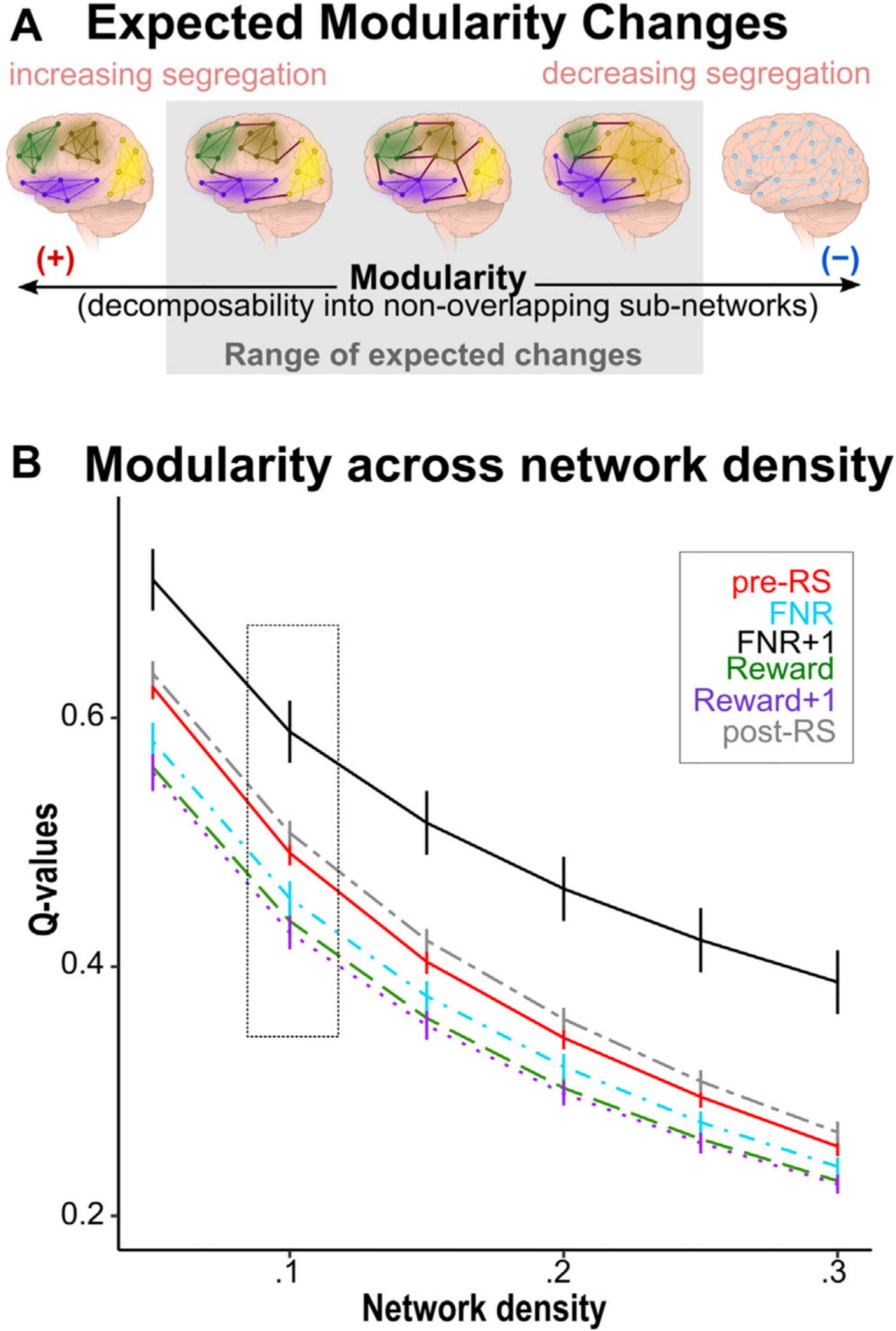
Changes in Modularity (Q) Throughout the Paradigm **Note:** (A) Range of potential changes in the modular structure of the brain during frustration. Depending on environmental events or internal states, the brain can transition to a more localized, segregated processing mode (left side of figure) characterized by clearly separable subnetworks with many within-subnetwork, and a few between-subnetwork, connections. Alternatively, the brain can transition to a more integrated processing mode (right side of figure). (B) During Reward, Reward+1, and FNR, the brain transitioned to a more global processing mode relative to the pretask resting state. However, during FNR+1 and the posttask resting state, functional segregation of the brain was higher than in the pretask resting state. This pattern is present across network densities, as shown on the x-axis. post-RS = posttask resting state; pre-RS = pretask resting state.

Aim 1 was to study brain network reconfiguration in response to frustration. First, we focused on modularity, or how well the brain can be subdivided into nonoverlapping groups of nodes (ie, modules) (Figure 2A). Modules are characterized by a high number of intramodule connections and a low number of intermodule connections.^38^ We calculated the modularity index (*Q*),^39^ which quantifies brain network segregation (ie, the extent to which the brain exhibits a modular structure), for each condition, estimated using the Louvain greedy algorithm^40^ implemented in the Brain Connectivity Toolbox.^17^ Higher *Q* values indicate higher segregation of the brain associated with more localized information processing, while lower *Q* values can be interpreted as more integrated information processing (Figure S2, available online). Given the stochastic initialization of the greedy optimization, it was applied 1,000 times for each condition. The highest *Q* value was used to compare modularity across conditions with paired *t* tests using 5,000 permutations, threshold *p* < .05 applying familywise error rate (FWER) correction across density thresholds (5%, 10%,15%, 20%, 25%, 30%),^41^ and Hedges *g* effect size.

### Does Frustration Impact the Structure of Specific Brain Networks?

Second, for each condition, we characterized the nodal composition of the modules present during that condition. Multiple module partitions maximize *Q*; thus, we used a consensus approach to calculate an agreement matrix across the 1,000 iterations for each participant and condition. For each condition, we calculated a matrix reflecting the probability of nodes being assigned to the same module across participants^37^ and subjected these matrices to the same community detection algorithm used at the individual level. We used the VIn metric^42^ to quantify the degree of dissimilarity in modular composition across the conditions. Significant differences in modular structure were determined using a repeated-measures permutation procedure with 5,000 permutations.^37,43^ To determine the contribution of specific modules to significant overall reconfiguration, we compared the VIn values for each module between conditions using paired *t* tests with 5,000 permutations.^37^

### Does Information Processing Efficiency of Specific Networks During Frustration Predict Irritability?

Last, we tested whether E_glob_ of any module during any condition predicted youth- and parent-reported irritability symptoms as well as task performance and task-induced changes in frustration. Prior work associates E_glob_ positively with neurophysiological,^44^ cognitive,^37^ and emotional processes.^20^ It is defined as the inverse of the average path length between all nodes^45^ and indexes the capacity for parallel information processing within a module. We calculated E_glob_ within each module from the group-level modularity partition at 10% network density. We used a prediction framework, dividing the sample into training/ validation and held-out/testing subsets (n = 48/n = 18) using stratified random sampling. All variables were normalized before the analysis. Predictors comprised the efficiency of all modules during all conditions, mean framewise displacement for all conditions, age, sex, medication load, and scanner. In the training/validation dataset, predictors were selected using linear stepwise regression, applying a 10-fold cross-validation with 20 repeats as implemented in the caret package for R (R Foundation for Statistical Computing, Vienna, Austria). The resulting model was used to predict youth- and parent-rated irritability, task performance, and change in frustration ratings in the held-out dataset. We used 5,000 permutations and a threshold of *p* < .05 applying false discovery rate (FDR) correction across the 5 models. Normal distribution of the residuals was confirmed by inspecting the *Q*-*Q* plots (Figure S3, available online). Correlation matrices for the criteria (youth- and parent-rated Affective Reactivity Index scores, increase in frustration, task behavior) and the efficiency of the modules during the different conditions can be found in Figures S4 and S5, available online. To determine specificity, we tested models for anxiety, inattention, and hyperactivity. A more detailed description of the methods can be found in Supplement 1, available online.

## RESULTS

### Does Frustration Impact the Degree of Brain Network Segregation?

Compared with pre-RS, the brain generally transitioned into a more global processing mode during the game 2 task events (FNR: *t*_65_ = −4.10, *p*_FWER_ < .001, Hedges *g* = −0.60; Reward: *t*_65_ = −6.51, *p*_FWER_ < .001, Hedges *g* = −1.04; Reward+1: *t*_65_ = −7.07, *p*_FWER_ < .001, Hedges *g* = −1.31). However, there was one exception: FNR+1 elicited more localized processing (*t*_65 =_ 6.93, *p*_FWER_ < .001, Hedges *g* = 1.36). Post-RS was also associated with more localized processing compared with pre-RS (*t*_65_ = 2.88, *p*_FWER_ = .008, Hedges *g* = 0.48), although the difference between pre-RS and post-RS was less pronounced than that between pre-RS and FNR+1 (Figure 2B; Figure S6, available online). There were no associations between modularity and motion (Table S1, available online). Associations between changes in *Q* and behavior and exploratory analyses regarding changes in *Q* and irritability are shown in Figures S7 and S8, available online.

### Does Frustration Impact the Structure of Specific Brain Networks?

**FIGURE 3:**
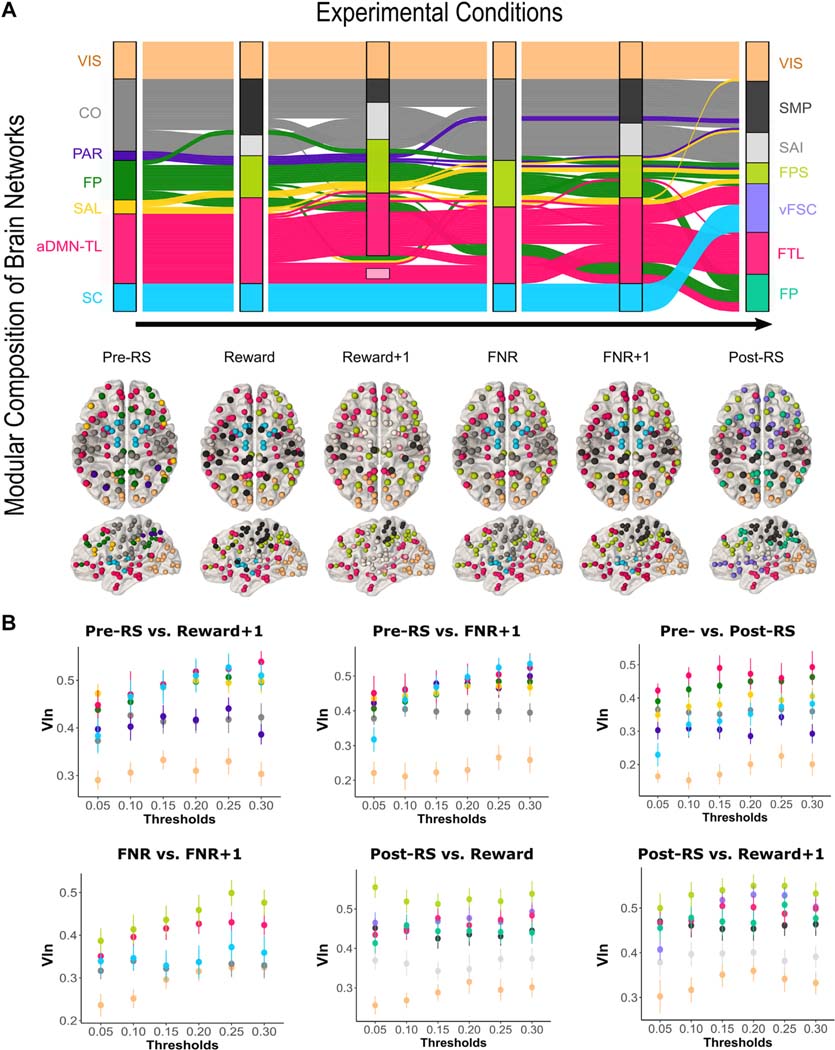
Modular Reconfiguration of the Whole-Brain Network During the 6 Experimental Conditions Note: (A) Alluvial diagram illustrates the reconfiguration of brain network modules from the pretask resting state (left) through the 4 task conditions (ie, Reward, Reward+1, FNR, FNR+1) and the posttask resting state (right). Modules identified in each condition are shown in the vertical boxes. The height of the boxes corresponds to the number of nodes within each module, and the streamlines depict how nodes originally belonging to one network change their membership throughout the paradigm. Below the flow diagram, nodes are overlaid on a brain template with colors corresponding to the module to which they belonged during that condition. During Reward+1, several nodes of the aDMN-TL and SC modules could not be assigned to modules with more than 3 nodes. (B) VIn values across the different modules for the task conditions, where significant differences between conditions were observed. The color scheme of the nodes is consistent with the scheme used in the alluvial plot for the first condition mentioned in the title of each plot. Error bars represent 95% CI. aDMN-TL = anterior default mode network–temporolimbic networks; CO = cingulo-opercular module; FNR = frustrative nonreward (ie, rigged feedback); FNR+1 = anticipation of new feedback after rigged feedback in previous trial; FP = frontoparietal module; FPS = frontoparietal-salience module; FTL = frontotemporal-limbic module; PAR = parietal module; post-RS = pos-task resting state; pre-RS = pretask resting state; Reward+1 = anticipation of new feedback after rewarding feedback in previous trial; SAI = somato-auditory-insular module; SAL = salience module; SC = subcortical module; SMP = somatomotor-parietal module; VIn = variation of information; vFSC = ventral fronto-subcortical module; VIS = visual module.

Here, we characterized the brain networks specific to each condition. In pre-RS, 7 modules were identified (Figure 3A). Based on the Schaefer atlas and previous literature,^36^ we labeled these modules as visual (VIS), cingulo-opercular (CO), parietal (PAR), frontoparietal (FP), anterior default mode network–temporolimbic networks (aDMN-TL), salience (SAL), and subcortical (SC) (Figure S9, available online). Modular composition differed significantly from pre-RS to FNR+1 (VIn = 0.18, *p* < .001), Reward+1 (VIn = 0.30, *p* < .001), and post-RS (VIn = 0.22, *p* < .001). Modular composition did not differ significantly between pre-RS and FNR (VIn = 0.12, *p* = .78), and differences between pre-RS and Reward (VIn = 0.16, *p* = .009) were unstable. Within the task, modular composition differed significantly between FNR and FNR+1 (VIn = 0.10, *p* = .002). See Table 1 for all results.

We next determined the contribution of specific modules to significant overall reconfiguration.^37^ Here, a relatively higher VIn value indicates a greater contribution of that module to overall reconfiguration. aDMN-TL and FP showed the highest VIn values (Figure 3B), whereas VIS showed the lowest VIn values (all *p*_FWE_ < .0001) (Figure 3B). Comparing pre-RS with both FNR+1 and Reward+1, aDMN-TL (all *p*_FWE_ < .0003) and FP (all *p*_FWE_ < .0072) showed higher VIn than CO. Differences between FNR and FNR+1 were also driven by aDMN-TL and FP, which showed higher VIn values than all other modules (all *p*_FWE_ < .0483). Last, differences between pre-RS and post-RS were driven by aDMN-TL and FP, which showed higher VIn than CO (all *p*_FWE_ < .0019), PAR (all *p*_FWE_ < .0399), and SC (all *p*_FWE_ < .0300) (Figure 3B).

Visual inspection yielded additional information about the reconfiguration of aDMN-TL and FP. During the task, one part of FP branched off to merge with aDMN-TL, while the remaining nodes of the original FP module were joined by nodes originally affiliated with PAR and SAL (Figure 3A).

During post-RS, 2 conjoined modules emerged. The ventral prefrontal nodes of aDMN-TL merged with SC to form a ventrofrontal-subcortical module (vFSC); henceforth, the remaining nodes of aDMN-TL will be referred to as the frontotemporal-limbic module (FTL). In addition, nodes originally affiliated with FP, PAR, and SAL merged to form a frontoparietal-salience module (FPS) (Figure 3A; Figures S10–S15, available online).

Given these results, we were interested in whether frustration per se drives the observed differences between pre-RS and post-RS modular composition. We reasoned that if modular composition did not differ significantly from FNR or FNR+1 to post-RS, but did differ significantly from Reward and Reward+1 to post-RS, this would provide some evidence that the frustrating task events are driving the observed differences between pre-RS and post-RS. Indeed, there were no significant differences in modular composition from either FNR (VIn = 0.26, *p* = .525) or FNR+1 (VIn = 0.23, *p* = .960) to post-RS, whereas there were significant differences from Reward (VIn = 0.26, *p* = .005) and Reward+1 (VIn = 0.36, *p* < .001) to post-RS, the latter of which were driven by changes in FPS (all *p*_FWE_ < .0493) (Figure 3B). This lends support to the hypothesis that frustration drives differences between pre-RS and post-RS.

### Does Information Processing Efficiency of Specific Networks During Frustration Predict Irritability?

**FIGURE 4:**
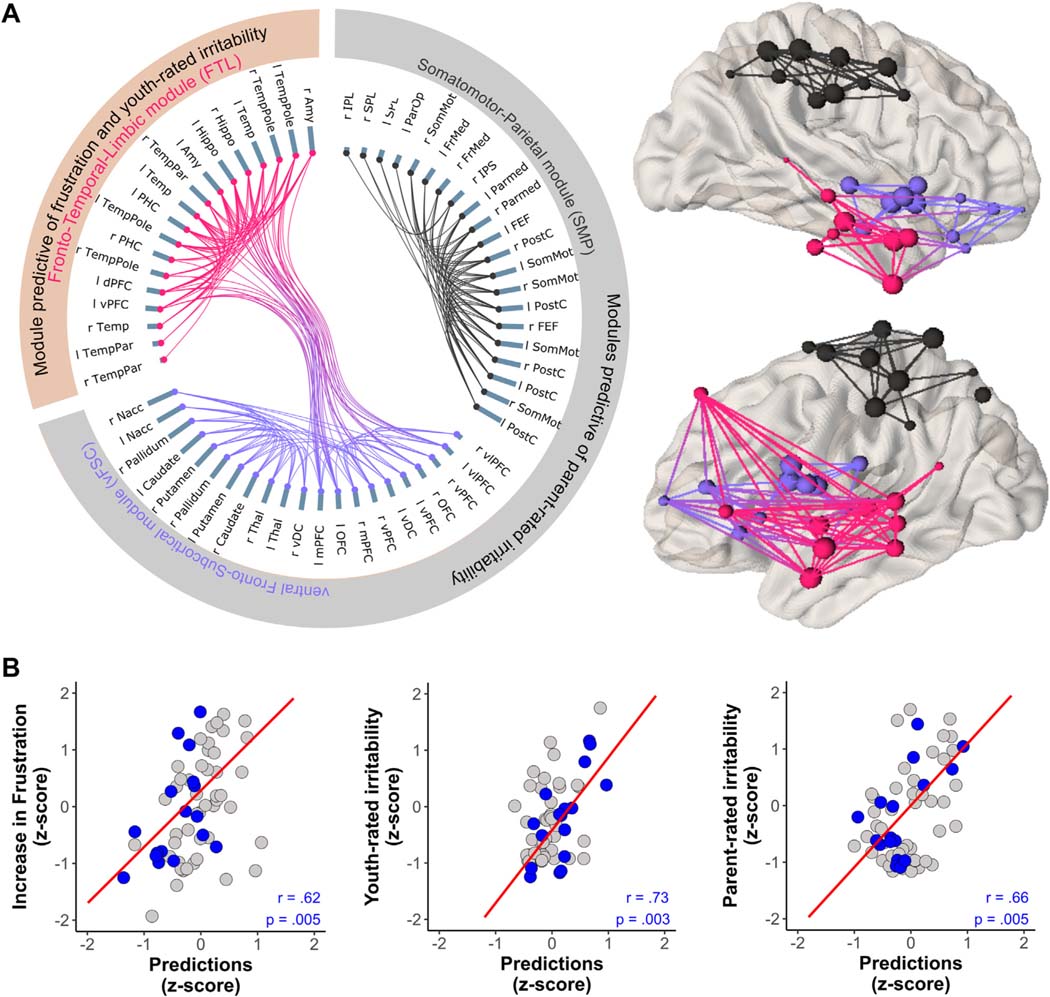
Module Efficiency as Predictor of Frustration, Youth-Rated Irritability, and Parent-Rated Irritability **Note:** (A) Modules present during the posttask resting state that predicted either increase in frustration and youth-rated irritability (pink frontotemporal-limbic module) or parent-rated irritability (gray somatomotor-parietal and lilac ventral fronto-subcortical module modules). The length of the gray-blue bars for each node represents the centrality of this node within the module, a graph-theoretical measure of the closeness of this node relative to all the other nodes in this module. (B) Separate graphs illustrate associations between the predicted increase in frustration, youth-rated irritability, and parent-rated irritability based on the significant predictors identified in the training subset vs the actual values in the held-out dataset (blue dots). Pearson correlation coefficients and corrected p values are also shown. This is overlaid on the predictions of the regression model for the training/validation dataset (gray dots). Amy = Amygdala; dPFC = dorsal prefrontal cortex; FE= frontal eye field; FrMed = medial frontal cortex; Hippo = hippocampus; IPL = inferior parietal lobe; IPS = intraparietal sulcus; L = left; mPFC = medial prefrontal cortex; Nacc = nucleus accumbens; OFC = orbitofrontal cortex; ParMed = medial parietal cortex; ParOper = parietal operculum; PHC = parahippocampal cortex; R = right; SomMot = somatomotor cortex; SPL = superior parietal lobe; Temp = temporal; TempPar = temporoparietal region; TempPole = temporal pole; ventral DC = ventral diencephalon, which includes hypothalamus, mammillary bodies, subthalamic nuclei, substantia nigra, red nucleus, and medial and lateral geniculate nuclei; vPFC = ventromedial prefrontal cortex; vlPFC = ventrolateral prefrontal cortex.

In the training/validation dataset, post-RS FTL E_glob_ (*β* = −0.25) and medication load (*β* = 0.38) predicted youth-rated irritability; these were also predictive in the held-out dataset (*r* = 0.73, *p*_FDR_ = .003, *R*^2^ = 0.53, root mean square error [RMSE] = 0.73) (Figure 4). Post-RS SMP (*β* = 0.38) and vFSC (*β* = 0.40) (Figure 4) E_glob_ predicted parent-rated irritability; these were also predictive in the held-out dataset (*r* = 0.66, *p*_FDR_ = .005, *R*^2^ = 0.44, RMSE = 0.82).

In addition to irritability, increase in frustration could be predicted from baseline frustration ratings (*β* = −0.29), Reward VIS (*β* = −0.28) and aDMN-TL E_glob_ (*β* = −0.23), and post-RS FTL E_glob_ (*β* = 0.25; *r* = 0.62, *p*_FDR_ = .005, *R*^2^ = 0.38, RMSE = 0.81) (Figure 4). Reaction time difference between valid and invalid trials was predicted by age (*β* = 0.41) and pre-RS (*β* = −0.29) and FNR+1 (*β* = 0.41) FPS E_glob_ (*r* = 0.59, *p*_FDR_ = .011, *R*^2^ = 0.35, RMSE = 0.93), while age (*β* = 0.34) and FNR+1 aDMN-TL (*β* = −0.29) and SC (*β* = 0.26) E_glob_ predicted accuracy (*r* = 0.46, *p*_FDR_ = .054, *R*^2^ 0.21, RMSE = 1.01). Models predicting symptoms of anxiety, inattention, and hyperactivity and task-induced changes in sadness in the training/validation subset did not show utility in the held-out dataset (all *p*_uncorrected_ > .13).

### Task Performance, Frustration Ratings, Pulse Rate, and Irritability

As expected, invalid cues were associated with longer reaction time (*F*_1,63_ = 20.66, *p* < .001, η^2^ = 0.25) and lower accuracy (*F*_1,63_ = 22.41, *p* < .001, η^2^ = 0.27). The introduction of FNR (game 2) was associated with faster responses during valid trials (*F*_1,63_ = 9.60, *p* = .003, η^2^ = 0.13), more errors during invalid trials (*F*_1,63_ =7.95, *p* = .006, η^2^ = 0.11), and increased frustration compared with game 1 (*F*_1,63_ = 18.26, *p* < .001, η^2^ = 0.23) (Figure 1C).

Pulse rate was best modeled as a quadratic function of time (ie, peaking during the task) with subject as a random effect. Because age (*β* = −0.99, *t*_59.2_ = −2.15, *p* = .036) and parent-rated irritability (*β* = 3.66, *t*_56_ = 2.83, *p* = .006) predicted pulse rate, they were included as fixed effects. Steeper pulse rate increase during the task was associated with more errors on invalid trials (*F*_2,52_ = 3.33, *p* = .001, η^2^ = 0.11).

## Discussion

In this pilot study, we used a frustration task flanked by resting-state scans to study brain reconfiguration associated with frustration. Here, brain reconfiguration refers to the modular reorganization that occurs as connectivity between nodes (brain regions) changes in response to the cognitive and emotional demands of the frustration task. Indeed, we found significant reconfiguration, consistent with the conceptualization of frustration as a potent emotional stimulus. Across conditions, reconfiguration of modules was driven by nodes originally affiliated with the FP and aDMN-TL modules, ie, frontoparietal, default mode, and limbic nodes. Multiple studies^37,46^ suggest that these regions play a crucial role in adapting to environmental demands. Our most clinically relevant analyses tested whether E_glob_, representing information processing capacity of brain modules, predicted the child’s irritability symptoms during the week before scanning. We found that E_glob_ of 3 modules predicted irritability. Importantly, all 3 predictive modules were present only in the posttask resting state, suggesting that maladaptive recovery from frustration plays a central role in the pathophysiology of irritability. Also notably, each of the 3 predictive modules mediates a process highly relevant to irritability, ie, emotion processing, reward processing, or motor behavior. We did not find any module in any condition that predicted anxiety, inattention, or hyperactivity, suggesting that the frustration paradigm is able to elicit brain responses specific to irritability. Given the study’s sample size, all of these findings require replication and must be considered preliminary.

At baseline (ie, in the pretask resting state), the module whose nodes would ultimately play a significant role in the prediction of youth-rated irritability (ie, aDMN-TL) included nodes in the temporal lobe (ie, temporal cortex, bilateral hippocampus, and amygdala), ventral prefrontal cortex, and anterior medial prefrontal cortex. Over the course of the paradigm, some nodes from this large module broke off to form the separate FTL module. Specifically, the FTL module present in the posttask resting state included bilateral amygdala, hippocampus, temporal cortex, and anterior medial frontal nodes and thus represented a core circuit for emotion regulation.^47^ E_glob_ in the FTL module negatively predicted child-rated irritability, suggesting that decreased information processing capacity in this limbic module could be associated with decreased emotion regulation after frustration and an increase in the child’s experience of irritability.

Similar to the FTL, the vFSC module also emanated from the pretask resting-state aDMN-TL and predicted irritability (in this case, parent-rated) in the posttask resting state. Specifically, the vFSC consisted of ventral and orbital prefrontal nodes that broke off from the aDMN-TL and joined with a pretask resting-state subcortical module that included basal ganglia nodes. E_glob_ in this vFSC module, which thus consisted of regions associated with reward processing, positively predicted parent-rated irritability. Frustration is the response to the omission of an expected reward; speculatively, increased efficiency in the vFSC may mediate a child’s exaggerated response to frustration that is observed by parents and contributes to the parent’s rating of increased irritability.

E_glob_ in the SMP module during the posttask resting state also positively predicted parent-rated irritability. FNR responses of this network have been previously associated with youth-rated irritability,^13^ and structural abnormalities in motor circuits have been associated with both parent and youth irritability ratings.^48^ Discrepancies between parent and youth irritability ratings are well documented.^49^ Evidence suggests that each informant captures unique aspects of the phenotype that are grounded in neurobiology.^50^ Further research is needed on mechanisms mediating informant effects, including studies such as this one that map ratings by different informants onto brain function.

While clearly preliminary, our findings provide important information that could guide the development of novel interventions. First, while we tested the predictive power of modules present in all paradigm conditions, all 3 predictive modules were present only in the posttask resting state, ie, the recovery period after frustration. Thus, our work highlights the potential importance of this period as a therapeutic target of treatments for pediatric irritability. In this regard, it is broadly consistent with 3 prior studies that found associations between irritability and neural activity after a frustrating event^12,14^ or task.^15^ In our data, the uniqueness of the post-frustration period is also supported by its relatively high *Q* value, which suggests a clearer division into distinct subnetworks during this period and more localized information processing. Future work could focus on the efficacy of therapeutic techniques designed to improve recovery from frustration in diminishing a child’s irritability.

Second, our work implicates 3 specific circuits that could be the target of novel interventions. These are a temporal circuit (FTL) mediating emotional responses, a ventral prefrontal subcortical circuit (vFSC) mediating reward processing, and a somatomotor-parietal circuit (SMP) mediating motor responses.

It is important to note the limitations of the present study. First, a clear limitation is the small sample size, raising concerns about replicability and generalizability. To mitigate this, in aim 2, we used a multivariate approach in combination with a train/test/held-out procedure instead of simple correlations. Nevertheless, replication in a larger sample is warranted. Such future work will be facilitated by multisite collaborations focused on phenotypes such as irritability for which frustration is a clinically relevant evoked state. Second, it will be interesting to see whether weak effects, such as the shift toward higher modularity during the recovery from frustration, are also observable in a sample that is not enriched for irritability. In such a sample, researchers could also test whether responses to frustration vary with cultural, ethnic, or environmental variables or with past experiences such as trauma. Third, future work, particularly work aimed at exploring clinical utility of these findings, should explore other selection techniques, such as least absolute shrinkage and selection operator or ridge regression, as stepwise regression does not necessarily identify the best set of predictors.^51^ Fourth, we did not include a control session to rule out the possibility that pre-vs post-task resting-state differences relate to the attentional aspect of the task or the passage of time, rather than to frustration. Our preliminary findings suggest that our predictions are specific to irritability vs other measures of psychopathology. However, given the sample size, this null finding might represent a type II error. Hence, follow-up research with a control task and larger sample is needed. Finally, we did not obtain frustration ratings after posttask resting state and thus cannot know whether the observed changes relate to the continued experience of frustration.

In sum, these pilot data suggest that frustration induces brain network reconfiguration driven largely by prefrontal, temporal, parietal, and limbic nodes. This reconfiguration persists into the post-frustration recovery period. Moreover, the capacity of brain networks for parallel information processing during this recovery period predicts individual differences in irritability, with preliminary data suggesting that this prediction may be specific. Predictive modules include nodes that mediate emotion regulation, reward processing, and motor activity. Novel interventions might focus on the post-frustration recovery period, and dysfunction in these brain networks may be a potential treatment target for youth with clinically impairing irritability. Given the small sample size, these findings should be considered preliminary until replication in a larger sample.

## Supplementary Material

MMC1

## Figures and Tables

**TABLE 1:** Variation of Information Statistics for the 6 Experimental Conditions Across Levels of Network Density

	Network density											
	
	5%		10%		15%		20%		25%		30%	
						
	VIn	*p*	VIn	*p*	VIn	*p*	VIn	*p*	VIn	*p*	VIn	*p*
Pre-RS												
Reward	0.131	> .999	0.155	.009	0.188	.014	0.203	.162	0.257	< .001	0.261	.194
Reward+1	0.189	.064	0.300	< .001	0.284	< .001	0.290	< .001	0.268	< .001	0.293	.002
FNR	0.175	.869	0.115	.777	0.115	.677	0.122	.809	0.195	.313	0.212	.822
FNR+1	0.162	.973	0.176	< .001	0.206	.004	0.248	.004	0.250	< .001	0.291	.002
Post-RS	0.134	> .999	0.223	< .001	0.197	.001	0.197	.006	0.203	.006	0.262	.002
Reward												
Reward+1	0.142	> .999	0.240	< .001	0.181	.341	0.191	.319	0.164	> .999	0.121	> .999
FNR	0.138	> .999	0.110	.918	0.111	> .999	0.144	.797	0.133	> .999	0.156	> .999
FNR+1	0.110	> .999	0.122	.061	0.172	.281	0.112	.990	0.158	> .999	0.143	> .999
Post-RS	0.187	> .999	0.260	.005	0.286	< .032	0.293	< .001	0.329	< .001	0.322	< .001
Reward+1												
FNR	0.146	.209	0.232	< .001	0.241	< .001	0.192	.062	0.194	.464	0.179	.515
FNR+1	0.126	> .999	0.202	.004	0.183	.763	0.140	> .999	0.129	> .999	0.105	> .999
Post-RS	0.277	< .001	0.358	< .001	0.336	< .001	0.336	< .001	0.349	< .001	0.327	< .001
FNR												
FNR+1	0.173	.007	0.103	.002	0.174	.005	0.164	.003	0.151	.006	0.164	.009
Post-RS	0.222	.838	0.260	.525	0.216	.817	0.207	.749	0.224	.603	0.295	< .001
FNR+1												
Post-RS	0.241	.196	0.232	.960	0.223	.397	0.256	.270	0.285	.042	0.244	.565

**Note:** FNR = frustrative nonreward operationalized as rigged feedback; FNR+1 = anticipation following rigged feedback; Reward = winning $0.50 after a correct response; Reward+1 = anticipation following a win of $0.50; RS = resting state; VIn = variation of information.
